# Label-free time- and space-resolved exometabolite sampling of growing plant roots through nanoporous interfaces

**DOI:** 10.1038/s41598-019-46538-5

**Published:** 2019-07-16

**Authors:** Damith E. W. Patabadige, Larry J. Millet, Jayde A. Aufrecht, Peter G. Shankles, Robert F. Standaert, Scott T. Retterer, Mitchel J. Doktycz

**Affiliations:** 1 Biosciences Division, Oak Ridge National Laboratory, PO Box 2008 MS 6445, Oak Ridge, TN 37831-6445 USA; 2 The Center for Nanophase Materials Sciences, Oak Ridge National Laboratory, PO Box 2008 MS 6445, Oak Ridge, TN 37831-6445 USA; 3 The Bredesen Center, University of Tennessee-Knoxville, Knoxville, TN USA; 4 Shull Wollan Center, Oak Ridge National Laboratory, PO Box 2008 MS 6445, Oak Ridge, TN 37831-6445 USA

**Keywords:** Analytical biochemistry, Nanofluidics

## Abstract

Spatial and temporal profiling of metabolites within and between living systems is vital to understanding how chemical signaling shapes the composition and function of these complex systems. Measurement of metabolites is challenging because they are often not amenable to extrinsic tags, are diverse in nature, and are present with a broad range of concentrations. Moreover, direct imaging by chemically informative tools can significantly compromise viability of the system of interest or lack adequate resolution. Here, we present a nano-enabled and label-free imaging technology using a microfluidic sampling network to track production and distribution of chemical information in the microenvironment of a living organism. We describe the integration of a polyester track-etched (PETE) nanofluidic interface to physically confine the biological sample within the model environment, while allowing fluidic access via an underlying microfluidic network. The nanoporous interface enables sampling of the microenvironment above in a time-dependent and spatially-resolved manner. For demonstration, the diffusional flux through the PETE membrane was characterized to understand membrane performance, and exometabolites from a growing plant root were successfully profiled in a space- and time-resolved manner. This method and device provide a frame-by-frame description of the chemical environment that maps to the physical and biological characteristics of the sample.

## Introduction

Metabolite tracking is key to understanding intracellular processes and functional events occurring at different biological hierarchies^1^. Metabolites are small molecules that serve as the currency of cellular systems to fuel their growth and facilitate communication. Observing their occurrence and location, particularly at cellular scales, represents a significant challenge. Compared to macromolecules, the small size and varied nature of metabolites prevent labeling with extrinsic tags, and their relatively rapid transit and broad concentration ranges challenge analytical measurement and imaging technologies. Current tools for characterizing metabolites are limited in their ability to spatially and temporally image metabolites in living systems and to identify the range and concentration of molecules that are present. Therefore, new approaches are essential for understanding the time-dependent spatial distribution of metabolites and for connecting their occurrence with phenotypic events beyond the cell.

Several metabolite imaging approaches have been developed. Genetically-encoded Forster resonance energy transfer (FRET) sensors provide one way to examine dynamic changes in the location of targeted metabolites^2,3^. This optical imaging-based approach is powerful; however, each FRET probe requires development and is limited to monitoring only one or a few intracellular metabolites concurrently. Fluorescence and vibrational spectroscopy techniques, including infrared and Raman, allow direct chemical imaging, however the identification of metabolites can be challenging^4,5^. Current approaches for comprehensive metabolite identification primarily use mass spectrometry (MS) or nuclear magnetic resonance (NMR) spectroscopy^1^. Mass spectrometry, often coupled with various chromatographic separation techniques, is most frequently used due to its sensitivity and suitability for identification and quantitation of metabolites^6^.

Spatially-resolved biochemical profiles can be measured by either spatially-resolved sampling or mass spectrometry imaging (MSI) techniques. MSI is a growing field with increasingly fine resolution but has continuing challenges in quantitation and in sampling biological systems in their native, live, hydrated state^7^. Most MSI techniques are implemented under vacuum, compromising the endogenous state, although not-yet commercially available techniques enable ambient ionization. Time-dependent biochemical changes can be measured by time course sampling and metabolic flux analysis (MFA), often using stable isotope tracers to measure flux through a biological system^8,9^. An ideal metabolite measurement technique would permit comprehensive identification of metabolites, quantitative measurement of concentrations, resolution on relevant biological scales, and real time monitoring, all in a native or near-native environment.

**Figure 1:**
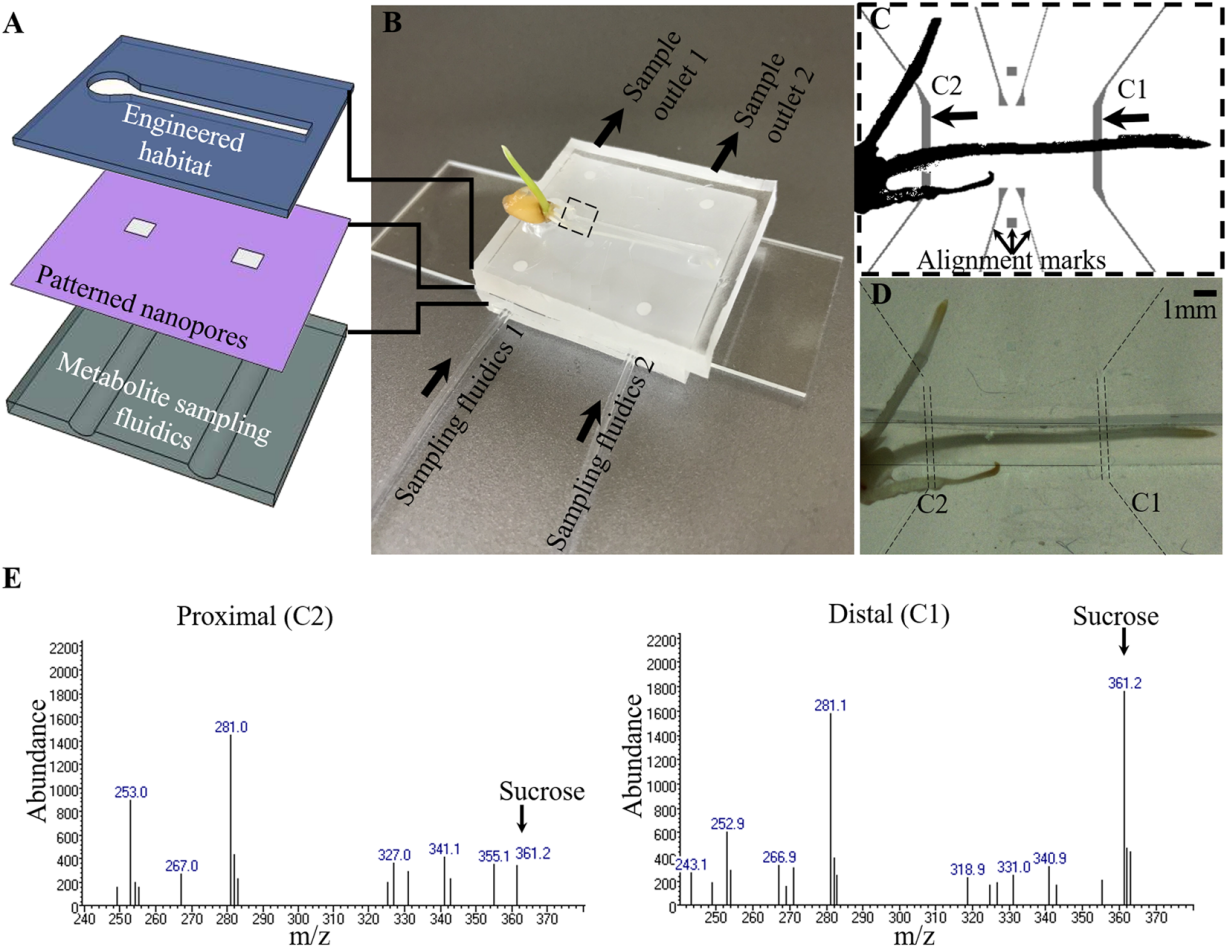
Microfluidic device for simultaneous imaging of live root development and for metabolite sampling. (**A**) Schematic showing assembly of the three layers used to produce a culture system for a growing wheat root, infusion-patterned nanoporous membrane for sampling, and sample collection for analysis of metabolites. (**B**) Photograph of a germinated wheat seed growing in the device. (**C**) The primary root is placed in the sample microenvironment layer. The underlying sampling channels (C1 and C2) allow fluid pumping through ports, enabling buffer exchange and sampling of metabolites at two individually accessible locations. (**D**) Brightfield image showing the plant root in the primary microchannel after 6 h of growth in the device. The underlying metabolite sampling channels, highlighted by dashed lines, are obscured by the sandwiched nanoporous membrane. (**E**) Mass spectra derived from extracted-ion chromatogram (XIC) of sucrose indicating differential levels of sucrose in samples collected proximal and distal to the seed. The trimethylsilyl derivative of sucrose elutes at 15.09 min under the given separation conditions. The sucrose chromatographic peaks were determined by aligning with the given XIC for a sucrose standard. The Student t-test indicates that sucrose produced at the C1 and C2 locations are significantly different at P < 0.05 (95% confidence interval).

Nanofluidic sampling presents the opportunity to characterize living systems in a minimally perturbing manner. Nanofluidic interfaces offer a means to spatially and temporally sample the local environment for downstream chemical analyses^10^. For measurements with high spatial, temporal, and chemical resolution, microsampling techniques combined with off-line analysis^11–14^ could be utilized but are invasive^15^. Here, we present a multilayered microfluidic structure for time-dependent, two-channel collection of chemical information from a growing plant root (Fig. 1). The device design provides an engineered habitat that allows simultaneous optical imaging of the system and time-dependent metabolite sampling from defined locations. Local sampling is achieved through patterned nanoporous membrane regions underneath the biological sample. Metabolites diffuse into and collect within an underlying set of microfluidic channels without introducing flow or mechanical disruptions to the biological sample. The diffused metabolites can be collected from individual channels at defined time intervals to provide time-dependent chemical information that correlates to specific regions of the biological system. This nano-enabled system provides a path to chemical imaging of living systems using MS-based measurement capabilities for comprehensive quantitative chemical profiling with a commercially available mass spectrometer.

## Results and Discussion

### Membrane patterning and device construction

To pattern spatially defined nanoporous sampling regions, two fabrication approaches were evaluated. Both approaches employ commercially available nanoporous PETE membranes with defined pore sizes and densities. To mask distinct regions of the membrane, an infusion patterning approach that uses stamping to permeate PDMS into select membrane pores was developed (SI Fig. 1). The infusion patterning technique was found to be effective for patterning feature sizes >35 µm. Features smaller than 35 µm were not found to be reproducible and were often poorly demarcated. This was due to re-flowing of the PDMS after peeling off from the patterning stamp. Therefore, to create higher resolution features, a micropatterned gasket was used and found to be capable of defining membrane regions down to 10 µm. In this approach, the PETE membrane is masked with a PDMS layer that contains the pre-determined sampling areas. Compared to the infusion patterning technique, the use of the patterned gasket requires an additional layer of PDMS, but does not require careful alignment under a stereoscope.

Sealing of the patterned membrane between device layers is a key requirement that affects subsequent fluid handling and proper device function. Previous approaches to incorporating commercially available membranes into microfluidic layers involve several operational and fabrication challenges^16^. In particular, weak bonding between microfluidic layers and nano membranes can result in leaking. For example, previous efforts described the use of multilayer microfluidic layers for sample pre-concentration and separation applications that employed polycarbonate track etched (PCTE) membranes and plasma treatment to facilitate bonding between the layers^17^. Plasma treatment is as effective as PDMS layers and can also irreversibly bond other substrates such as glass, silicon and oxide layers^17,18^. However, plasma treatment causes oxidative damage to certain commercial membranes, irreversibly changing chemical properties. Infusion patterning eliminates plasma treatment as it allows wet PDMS to diffuse to both sides of the PETE membrane. This promotes subsequent, irreversible bonding to adjoining plasma treated microfluidic layers. Infusion pattering facilitates seamless integration of the membrane and eliminates issues with bonding and leaking. Further, the technique should be generally applicable for use with other membrane materials. The use of the patterned gasket serves as a powerful approach to fabricating small feature sizes down to 10 µm, but does require additional fabrication steps, and the bonding of PETE with the adjacent PDMS layers is reversible. The bonding strength can be improved by applying a thin film (~6 µm) of wet PDMS onto the surface of one of the PDMS layers. Nevertheless, both fabrication techniques allow the use of flow rates of up to 15 µL/min in the 20 mm × 200 µm × 20 µm channels without leakage.

### Chemical flux through the membrane

The structural design of the multilayered fluidic device allows the local chemical environment to be sampled through the nanoporous membrane. To test the efficacy of the device to transmit chemical information to the underlying sampling channels, and to quantify the effects of aperture size and sampling parameters, the flux of fluorescently labeled reporter molecules was examined. FITC and fluorescently-labeled dextran were used as small and large molecule surrogates, respectively. Molecular flux is expected to be governed by both the sampling window membrane properties (i.e., membrane thickness, aperture size, number of nanopores, nanopore radius) and the analyte properties (concentration gradient and diffusion coefficient). Using a common membrane structure, the membrane thickness (~10 µm) and the nanopore radius (200 nm) remain unchanged for any given experiment, while the number of nanopores depends on the pixel size of the sampling window (23, 48 and 100 µm). Based on the assumptions of Fickian diffusion through nanopores^19,20^, and an unchanging concentration gradient, the expected flux rates for FITC and dextran reporter molecules are summarized in Table 1 for three pixel sizes. For a 0.8 mM concentration gradient, small molecules like FITC move rapidly across the membrane, on the order of ~200 ms, with a molecular flux on the order of femtomoles per second. On the other hand, large molecules such as dextran, at a 0.1 mM concentration gradient, diffuse relatively slowly, on the order of ~2 s. The expected and measured fluxes for these model analytes were consistent (Table 1).

**Table 1:** Effect of aperture size on molecular flux of 0.8 mM FITC and 0.1 mM dextran through nanoporous membrane.

Pixel size (µm)	# of pores	FITC (MW = 389.38 Da), D = 5 × 10^−6^ cm^2^/s		Dextran (MW = 40 kDa), D = 5.1 × 10^−7^ cm^2^/s	
		Expected flux (femtomole/s)	Measured flux (femtomole/s)	Expected flux (attomole/s)	Measured flux (attomole/s)
23	415	2.0	1.8 ± 0.3	25.8	21.0 ± 0.6
48	1809	8.8	7.6 ± 1.6	112.4	84.4 ± 7.5
100	7853	38.3	30.6 ± 4.8	488.0	303.8 ± 2.6

Note: The measured average flux was calculated using the measured flux at each time point (i.e., 5, 10, 15, 20, 25, 30 and, 35 min for FITC diffusion, and 20, 30, 40, 50, 60, and 70 min for dextran diffusion).

**Figure 2:**
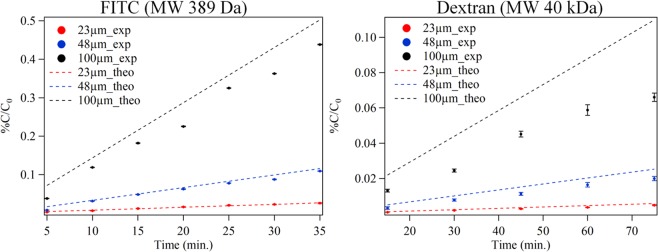
Effect of pixel size on the rate of diffusion of (**A**) FITC, a small fluorescent molecule and (**B**) labelled dextran, a large fluorescent molecule. Diffused fraction (%C/C_0_) with respect to sampling time in stopped-flow mode. Solid points indicate experimental values, dashed lines indicate linear regression results.

A flux time course was conducted with each model analyte using the multilayer fluidic device in a simple cross layout with a sandwiched membrane. Flux values were determined by collecting the analyte in the underlying sampling channels in a “stopped-flow” mode where diffused analytes were allowed to collect in the sampling channel and were then extracted after a defined time period. This approach reduces the collection volume and increases the analyte concentration relative to operation in a continuous flow mode. The experimentally determined flux, as measured by the relative concentration of the collected material (%C/C_0_; percent concentration present in the collection channel relative to the original analyte concentration in the sample chamber), shows a linear relationship with collection time and agrees with the expected values based on Fickian diffusion (see Fig. 2). Excellent fits are observed for FITC diffusion, especially at short time periods and small sampling aperture sizes. Larger pixel sizes and longer collection time periods result in slightly increased deviations. This is likely caused by the build-up of analyte in the collection channel that alters the actual concentration gradient compared to the constant gradient used in the analytical model. The differences in the calculated and experimentally measured diffusional flux for dextran show even greater deviations, especially for the larger pixel sizes and longer time periods. This is likely due to increased molecular interactions of dextran with the membrane compared to FITC. Surface fouling, clogging and adsorption to the membrane and PDMS surfaces are expected to increase for large molecules^16,17^.

These experimental parameterizations highlight device characteristics that can be optimized for particular applications and guide the design of time- and space-resolved exometabolite sampling. The simple model of Fickian diffusion adequately predicts the chemical flux behavior for both small and large molecules. These predictions provide better estimates for small molecules and under the conditions where the concentration gradient remain constant. In general, chemical flux through the membrane increases with sample aperture size and the number of nanopores. This allows for rapid sampling but at the expense of spatial resolution and potential perturbation to the sample environment. Under the examined conditions, the relative concentrations of collected material were all well below 1% and would be expected to minimally perturb biological systems. In practice, the spatially and temporally dependent detection of certain analytes will depend on their physical characteristics, environmental concentration, and detection efficiency and require optimization of the aperture size and/or device surface chemistry for particular applications.

### Detection of model plant root exudates

To evaluate the feasibility of monitoring plant root exudates in the multilayer fluidic device, the detection limits of expected metabolites were measured. Understanding these limits will depend on the relative concentration of the collected material (% recovery) and detection sensitivity for particular metabolites. Therefore, lower limits of detection were determined for a set of metabolites typically produced as root exudates by growing plants using standards ranging in concentration between 1 µM–3.2 mM^21^. Based on the previous results with fluorescently labeled molecules, this micromolar concentration range would be diluted to nanomolar levels after passing through the nanoporous membrane. Mixtures of analytes were introduced into the sample culture fluidic layer, and samples were collected using the stopped-flow mode after 8 h. The collected samples were analyzed by GC-MS and compared to detection limits for the instrument determined from dilutions of the same standard mixtures and considering the smallest peak that could be integrated. The lower limits of detection (LLOD) for the given analytes in the metabolite sampling channels were in the range of ~1.5 nM to ~180 nM and are consistent with the sensitivity values obtained for the MS detector. Deviations can be expected due to sample preparation errors and loss of samples during pre-concentration and derivatization steps (Table 2). Amino acids such as alanine, isoleucine and valine could be detected in the low nM range whereas carboxylic acid derivatives such as malate were detectable at high nM levels in the metabolite sampling fluidic layer. Under these conditions and for these selected model analytes, estimated metabolite concentrations in the sample microenvironment in the range of 1.0 µM–0.21 mM would be required for detection (Table 2).

**Table 2:** Diffusion and detection of example root exudate metabolites through nanoporous membrane.

Metabolite	%C/C_0_ Fraction diffused^a^	LLOD (nM)	LLOD (µM)	LLOD (nM)
		Standard	Culture micro environment	Metabolite sampling layer^b^
Valine	0.19 ± 0.025	1.3	1.0	1.6
Alanine	0.16 ± 0.015	1.5	1.0	1.6
Isoleucine	0.14 ± 0.023	1.3	1.6	1.5
Phenylalanine	0.12 ± 0.013	11	10	11
Sucrose	0.10 ± 0.0047	27	48	42
Malic acid	0.10 ± 0.0018	1.4 × 10^2^	2.1 × 10^2^	1.8 × 10^2^

^a^%C/C_0_ Fraction diffused was determined from amount of materials present in the 20 µL of sample microenvironment and 20 µL metabolite sampling layer after 8 h of diffusion through 50 × 50 µm window.

^b^The detection limit of metabolite sampling layer corresponds to that of sample environment.

Though the examined standards are all small molecules, variable limits of detection are observed. In general, the relative fractions of the collected materials are in the range of 0.1–0.2%. As with FITC, the analysis of these selected analytes indicate that they undergo several hundred times dilution when diffusing from the sampling environment to the collection channel. Dilution depends on the pixel size and sample collection time. These analytes are all small hydrophilic molecules and differences in lower limits of detection are primarily due to ionization efficiency. However, differences could also arise from the effects of chemical properties on diffusion, volatility, derivatization efficiency, adsorption to the membrane, PDMS, sample vials, or pipette tips. These effects are more substantial at concentrations approaching the lower limit of detection. Quantitative analyses will need to rely of the use of external standards sampled from the device in matrix or internal standards with known or measured response factors that include the contribution from the sampling process. Nevertheless, the results suggest that typical concentrations of plant root exudates are within the detectable range and the platform can readily supply samples for analysis by GC-MS.

### Spatiotemporal tracking of plant root exudates

To examine the feasibility of dynamically monitoring plant metabolites in spatially defined regions, the local fluid environment around a growing wheat root was examined. The chemical environment formed in proximity to the plant root, exemplifies a system with a dynamic spatial metabolite distribution. Plant roots exude hundreds of different metabolites that serve to recruit and stimulate the growth of beneficial microorganisms and ward off pathogens, among other functions^22–24^. These specialized chemical profiles can include amino and other organic acids, sugars, phenolics, polysaccharides, proteins, and many metabolites with unknown function^22^. Typically, exudate materials and concentrations depend on the plant’s developmental stage and environment, including microorganisms that interact with the roots^25^. To track wheat root exudates, a seedling was placed in a sample microenvironment chamber and exudate production was monitored at locations near the germinated seed and near the growing root tip (8 mm apart) using 200 × 200 µm nanoporous sampling windows (Fig. 1). Analytes from these two locations were collected at two-hour increments over the course of eight hours using stopped-flow collection and analyzed by GC-MS. As a control, wheat seedlings were grown in microfluidic sample environments without the underlying membrane and metabolite sampling layers and samples were simply extracted from the same locations using a pipette. In all cases, the collected material was found to be composed of hundreds of unknown organic components as well as known materials including amino acids, sugars, carboxylic acids and their derivatives. The total collection of identified metabolites is provided in the supplementary information (SI Table 1). In general, the metabolites identified are consistent with previous studies carried out using other plant seedlings^23,26,27^. In contrast, these previous measurements were done using bulk analyses and performed on exudates collected over time periods of days and weeks.

Simple sampling using a pipette to collect liquid from locations near the root shows that a large number of known and unknown metabolites can be detected. These control experiments also show a general increase in analyte concentration with time but poor distinction between analyte concentrations in different locations (SI Fig. 3). Further, this sampling approach resulted in significant variations between measurements for particular metabolites but only slight variations in the concentration for other analytes. Overall, standard deviations increased for measurements at later time points. For example, at t = 6 h, the relative standard deviation for valine was 48.3% compared to 4.6% at the 2 h time point. These observations may be due partially to spatially dependent generation of metabolites and diffusive mixing of these materials in the plant growth environment. Although, these results also indicate that spatially dependent bulk sampling does not provide reliable and consistent measurements. Further, collecting these time and location dependent measurements required different plant root samples as aspiration in the sample environment completely depletes the hydroponic environment and disturbs the sample. Overall, bulk sampling approaches prevent spatiotemporal tracking of metabolites of a growing root.

**Figure 3:**
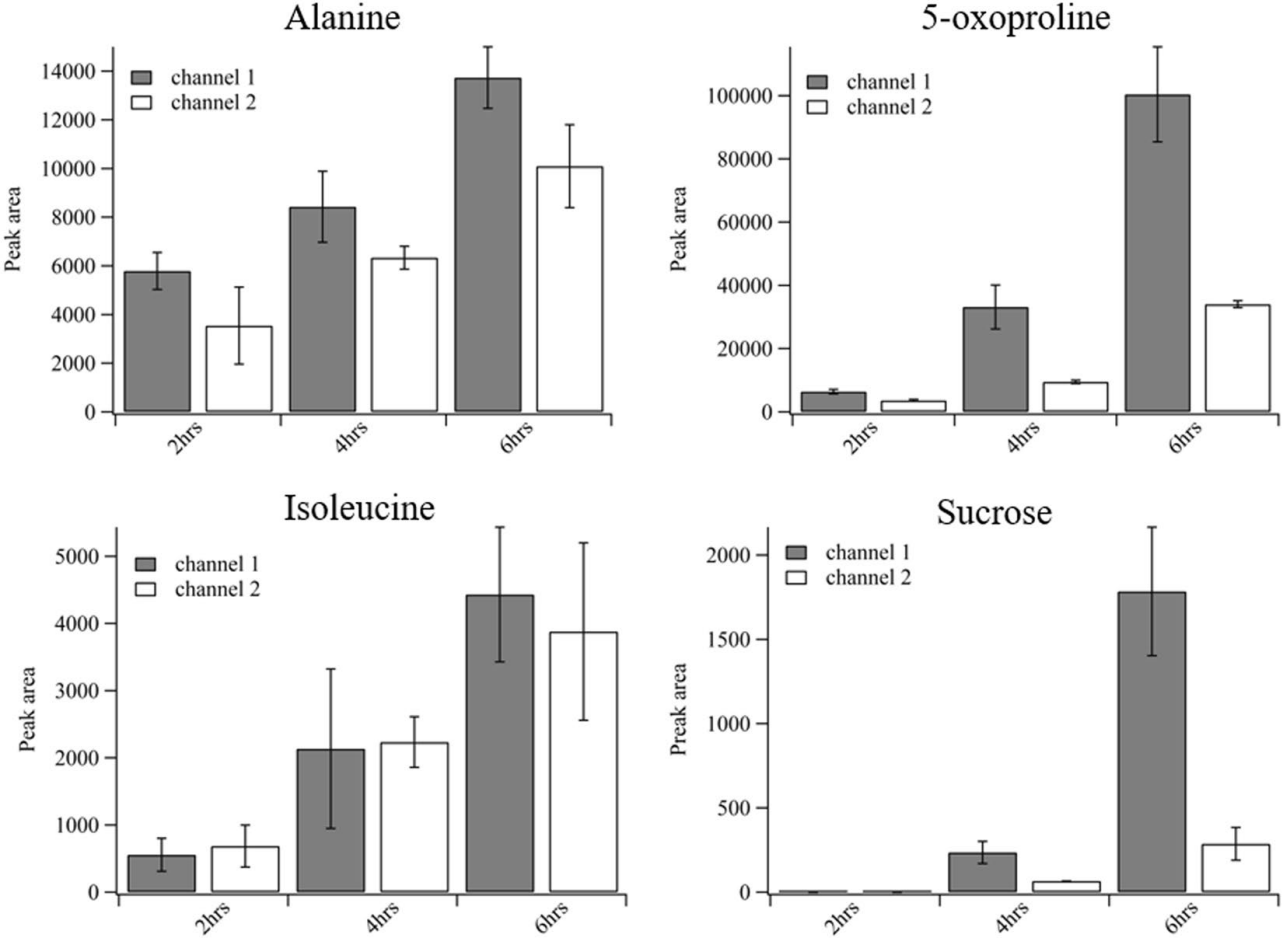
Concentration variation of selected metabolites over time with respect to two distinct locations close to root tip (channel 1) and root base (channel 2). Isolated metabolite flux was sampled using channel 1 and channel 2 respectively. Error bars represent the standard deviation of three experiments. The mean concentrations for all metabolites except isoleucine show statistically significant differences at the 95% confidence interval for the two sampling locations at the six hour time point. Statistically, except for 5-oxoproline, no significant difference was observed for alanine, isoleucine and sucrose peak areas at time points less than six hours.

Metabolite sampling using the multilayered fluidic device with defined nanoporous membrane regions produced clear differences in metabolite concentrations as a function of time and location. The local, diffusive based metabolite extraction allows isolation of the metabolite flux resulting from specific regions of the root. As shown in Fig. 3, for particular metabolites, the collected analytes vary depending on the different root locations that were sampled. Additionally, time dependent concentration differences are evident. Several amino acid derivatives were detectable at t = 2 h, however, sucrose was not detected until t = 4 h. Overall, metabolite concentrations increase over the course of experiment and more material is generated near the growing root tip. These spatially- and temporally-dependent metabolite concentration changes indicate that exudates are not uniformly produced along the length of the root. This uneven distribution of exudates is consistent with observations using biosensor strains to track the spatial distribution of sugar compounds and aromatic amino acids in the rhizosphere of *Avena barbata* roots^28^. Further, observations of *Arabidopsis thaliana* roots have also been shown to exude sugar compounds from the root tip and aromatic molecules at distal locations^28^. These differences in exudation profiles may lead to differences in microbial colonization patterns that have been observed within plant on a chip systems^29^.

The nanopore-based sampling technique is capable of tracking secondary metabolites in the sample microenvironment with good precision. Using the biological system and device design presented here, metabolites were reproducibly distinguished at a temporal resolution on the order of a few hours and spatial resolution on the order of a few millimeters. Low nanomolar concentration levels can be detected in the metabolite sampling channel and approaches to improve this sensitivity and the range of metabolites identified can be considered. Beyond the use of improved instrumentation, optimization of sample pre-treatment processes may extend detection limits for certain analytes. For example, plant root exudates typically consist of many sugars, including glucose, fructose, maltose, trehalose, and sucrose^21^. However, only sucrose was identified in these analyses. Using the present procedures, sugars may have precipitated during sample preparation steps, may not have derivatized completely with MSTFA due to competitive reactions of other metabolites (especially amino acids), or may have been present at levels below our limits of detection. Improvements to procedural aspects of the technology will likely extend the range of metabolites detected and improve the limits of detection. Evaluation of the derivatization efficiency using standard mixtures can enhance the accuracy of quantification.

The ability to integrate mass spectrometry-based analyses with the microfluidic-based sampling platform enables the collection of rich chemical information related to the biological system. Mass spectrometry-based chemical analysis techniques eliminate the need for molecular tagging and can greatly expand the number of metabolites that can be monitored when compared to spectroscopic approaches^30^. Extracting this chemical information through a nanoporous interface enables collection of this information on a living system in a non-destructive manner, in contrast to currently available mass spectrometry imaging techniques that are destructive to living systems^31^. Further, direct sampling techniques, as tested here, require significant collection of the surrounding fluid that compromises the local environment and measurement of temporally dependent changes.

Key attributes of the nanopore-based sampling approach are the adaptability of the platform and the ability to simultaneously optically image and chemically sample the system in real time. Custom definition of the sample environment and nanopore sampling locations allow modification of the platform to suit different biological systems and to detect particular analytes. Application to microbial biofilms and eukaryotic cell cultures are potential applications. Nanoporous interfaces with different physical and chemical characteristics, such as pore size, thickness and hydrophilicity, can be patterned to develop size exclusion based filtration techniques^17^. Additional sampling channels and sampling locations can be introduced to enhance the spatiotemporal imaging capability. Moreover, this device can be adapted to simplify fluid pumping and automate process steps by introducing on-chip microfluidic soft lithographic valves^32^. Such an approach could eliminate external tubing and mechanical pumps. Moreover, the microfluidic sample chamber can be adapted to analyze a variety of other plant systems, image microbial activity around the plant root^33^ and profile dynamic chemical changes over space and time.

The wheat seedlings examined here are relatively large and germinate quite rapidly compared to model plants that have been previously examined using bulk analysis techniques^23,26,27^. For example, *Arabidopsis thaliana* seedlings are considerably smaller and may take several days to weeks to observe significant growth or to collect metabolite profiles^23^. The ability to simultaneously image and collect metabolite information in real time allows correlation of chemical and physical information. For example, the wheat seedling’s root grew at an average rate of 170 ± 29 µm/h (for 3 trials) and the detected metabolites primarily emanated from the root tip.

## Conclusions

The described multilayer fluidics technology allows space-dependent tracking of the dynamic chemical environment surrounding living systems. This approach takes advantage of patterned nanopore membranes to facilitate chemical imaging in a minimally perturbing manner and can be customized for different biological imaging applications. By defining the size of the sampling aperture and the nanopore characteristics, diffusive flux for both small and large organic molecules through the membrane can be predictably defined. Metabolites collected through this membrane can be directly analyzed by mass spectrometry, and the detection limits are compatible with biologically relevant metabolite concentration levels. The system has been demonstrated for the mapping of plant root metabolites and the spatially and temporally dependent production of root exudates are shown to correlate with different regions of the growing root. This nano-enabled approach to sensitively detecting and measuring metabolites adds the needed chemical dimensions to optical imaging and can facilitate understanding of complex biological systems.

## Methods

### PDMS microfluidics

For preparation of microfluidic masters, bare, four-inch silicon wafers (WRS materials, San Jose, CA) were used as starting material. Wafers were coated with an adhesion promotor (MicroPrime P20, 3000 RPM, 45 s) and then coated with NFR photo resist (3000 RPM, 45 s). Coated wafers were baked (95 °C, 90 s), exposed (50 mJ/cm^2^), post-exposure baked (115 °C, 90 s) and developed (CD-26 developer) until the unpolymerized resist was cleared. Developed wafers were then rinsed with deionized (DI) water and dried with a nitrogen stream. The feature height produced by this protocol was ~2 µm.

To alleviate surface cracking and to ensure structures remained intact with the wafer, patterned wafers were etched using reactive ion etching such that the final feature height was 20 µm. These microfluidic masters were then coated with a layer of (trichloro(1H, 1H, 2H, 2H-perfluoro-n-octyl)silane, 85 °C, 60–120 min)) to prevent PDMS adhesion to the master. Microchannel replicates were molded from the wafer by casting PDMS (10:1, prepolymer:curing agent ratio) onto the wafer, degassing to remove bubbles, and baking (70 °C, 2 h). Cured PDMS was removed from the wafer, trimmed, punched with dermal biopsy punches, and cleaned with 3 M Scotch® Magic™ tape.

### Patterning nanoporous membranes

Two processes were devised to pattern nanoporous membranes with defined sampling areas. These processes achieve the same functionality but with different feature resolution. To define nanoporous windows that enable sampling through features >100 µm in length, infusion patterning with microcontact printing of PDMS was used. Nanoporous sampling windows with features ≤100 µm were achieved through the creation of a micropatterned gasket. In both cases, commercially available polyester tracked etched nanoporous membranes (90 mm diameter PETE membrane filters with 400 nm pores at a pore density of 2 × 10^6^ pores/cm^2^, PET049030, Sterlitech Corp.) were used (SI Fig. 1). Infusion-patterned nanoporous membranes were formed by coating a clean silicon wafer with a 10:1 liquid PDMS mixture at 5000 RPM for 15 min to produce ~6 µm PDMS film. A negative PDMS mold was then laid on the wet PDMS film. This PDMS mold was carefully lifted off and pressed against the PETE membrane. This technique allows transferring wet PDMS onto the PETE membrane, stamping micro features, and selectively blocking the majority of nanopores while keeping pre-determined sampling/dosing areas open (SI Fig. 1A). The PDMS stamp was carefully peeled off and the patterned membrane (wet PDMS facing up) was placed on the supporting blue paper provided by the vendor. The patterned PETE membrane was covered with a Petri dish and placed in a laminar flow hood to prevent the adhesion of dust particles onto the wet PDMS film. The PETE membrane was kept for ~2 h under ambient condition to diffuse the wet PDMS through the membrane. Afterwards, the supporting paper and the patterned membrane were then carefully transferred to a hot-plate (60 °C) to cure for 2 h.

Creation of micropatterned gaskets is an alternative technique that can be used in the fabrication process when chemical sampling windows with features <100 µm are needed. Briefly, ~6 µm thin porous PDMS membrane^34^ was sandwiched between the metabolite sampling channel and the un-patterned PETE membrane. This PDMS membrane requires a supporting layer, therefore, it was attached to the metabolite sampling-PDMS layer using a multilayer, soft lithographic technique (SI Fig. 1B)^32,35^. The creation of a porous PDMS membrane was based on a previously reported technique^34^. First, pentagonal features with diameters of 25, 50 and 100 µm were fabricated using a ~2 µm thin coated layer of NFR photo resist (3000 RPM, 45 s) on a clean silicon wafer. Then, the silicon wafer was etched using reactive ion etching (Oxford RIE, PlasmalabSystem 100, UK) to produce 70 µm high pentagonal posts (SI Fig. 1B)^34^. The post dimensions were measured using a stylus profiler (KLA Tencor P-6, CA) and surface characteristics were visualized and imaged by scanning electron microscopy (Carl Zeis Merlin SEM, Germany). Then, 10:1 PDMS was poured on this silicon wafer and spun at 5000 RPM for 15 min to produce ~6 µm thin film of PDMS such that silicon posts were standing above the PDMS liquid film (SI Fig. 1B). Finally, the supporting layer, or metabolite sampling fluidic layer, was attached as described above and pore dimensions were characterized with a stylus profiler and scanning electron microscope.

### Device operation

Externally driven syringe pumps were used to automate the fluid handling process using individually accessible metabolite sampling ports in the underlying metabolite sampling layer (Fig. 1B). Flow rates of 5 µL/min were used to flush fluids from the sampling channels and into collection vials for downstream analysis. To connect tubing to the sampling ports, a corner-punch hole was cored halfway through the PDMS from the top (perpendicular to the channel), followed by punching a second hole, in a parallel direction, to make a right-angle fluidic reservoir (Fig. 1B and SI Fig. 2). This tubing connection strategy from the sampling port to the external syringe pump causes minimum perturbation to the PETE membrane^36^.

### Device characterization by measurements of diffusion of fluorescent dyes

To assess diffusion and transport through the device, molecular diffusion between the layers and through the patterned membrane was measured using aqueous solutions (pH = 6.6) of 0.8 mM FITC and 0.1 mM dextran (M.W = 40 kDa) for representative small and large molecules, respectively. Molecular flux was measured using three sampling apertures, or “pixel sizes”, in which nanopores were uniformly distributed over 23, 48 or 100 µm-sized pentagonal areas. In each case, the top and bottom fluidic layer were arranged in a simple cross-shaped pattern. The top sample culture fluidic layer contained a 200 µm wide by 20 µm deep microchannel and was filled with the solution containing the fluorescently labeled probes. The diffused solution was collected after passing through the 10 µm thick patterned membrane and into the underlying 200 µm wide by 20 µm deep sampling channel by pumping DI water at a flow rate of 10 µL/min. Fluorescence of the collected samples was measured using a Perkin Elmer Multilabel Plate Reader (2300 EnSpire multilabel reader, Singapore) and quantified using standard calibration curves.

### Metabolite profiling of plant root exudates

For measuring metabolites emanating from a growing plant root, a wheat seed was first placed in a culture plate containing agar and liquid Murashige-Skoog media (hydroponic plant nutrient solution)^37^ and grow hydroponically as previously described^33^ for 40 h. A Percival Model I30BLL growth chamber (Percival Scientific, Perry Iowa) was set to 16 h dark/light cycle at 23 °C and culture plates containing seeds were placed vertically to promote “gravitropism growth”^29^. The seedling was then transferred to the sample microenvironment of the multilayered fluidic device and contained Murashige-Skoog media. This defined the experimental start time, t = 0. For these experiments, the dimensions of the sample environment primary microchannel were 2 mm × 3 mm × 2.5 cm. At t = 0, the total length of the plant root averaged 13.6 ± 2 mm for 3 trials. Two underlying, parallel sampling channels, 8 mm apart, comprised the metabolite sampling layer and were 200 µm wide by 20 µm deep (see Fig. 1). Sampling channel 1 was proximal to the germinated seed and ~2.8 mm away. Sampling channel 2 was ~10.8 mm distal from the germinated seed. The chemical environment generated at these two locations were collected through 200 µm × 200 µm square pixels patterned onto the PETE membrane. Three measurements were taken at 2 h increments by pumping (at the flow rate of 5 µL/min) DI water through the sampling channels. For reference, control experiments were designed in which the culture chamber was attached to a supporting PDMS layer without using the nanoporous membrane or underlying fluidics. In each case, a 20 ± 3 µL sample volume was directly collected from the culture chamber for analysis by gas chromatography-mass spectrometry (GC-MS).

### Metabolite analysis using GC-MS

Metabolites were analyzed by GC-MS using an Agilent 7890A GC and 5975C MSD quadrupole MS. Collected samples were first pre-concentrated and derivatized by transferring the materials to 150 µL deactivated (silanized) GC vials and evaporating the solvent using a vacuum evaporator at 35 °C for 20 min. Then, 25 µL of anhydrous acetonitrile was added to the GC vial for dissolving the organic components, followed by the addition of 25 µL derivatizing agent (N-methyl-N-(trimethylsilyl) trifluoroacetamide; MSTFA) to the solution. Next, the mixture was incubated at 70 °C for 1 h. The mixture was allowed to sit for 2 days at ambient conditions before analyzing with GC-MS. The derivatization efficiency was not calculated, and it was assumed that the 48 hour incubation with excess reagent brought the derivatization reaction to equilibrium. Then, 1 µL of sample was analyzed using an Agilent 5975 triple quadrupole GC-MS system with HP-5ms column (5% phenyl/95% dimethylpolysiloxane) with a 0.25 μm stationary phase, 30 m long separation column and 10 m long guard column. Splitless injection was used to inject 1 μL of sample each time. The oven temperature was kept at 50 °C for 2 min and then ramped at a rate of 20 °C/min to 325 °C and held at 325 °C for 11.5 min. The flow rate was set to 1 mL/min. The mass spectrometer was operated at EM voltage of 1118 V with the mass range of 50.0–650.0 Da. Mass spectra were set to record with a 6 min solvent delay. Compounds, except 5-oxoproline, were identified by comparison of retention times to those of standards. The 5-oxoproline derivative was identified by comparing the mass spectra of the 5-oxoproline-2TMS derivative to the National Institute of Standards and Technology (NIST: version 2017/2014/EPA/NIH) mass spectral library (SI Table 1). The Student t-test was performed to evaluate statistical significance (at 95% confidence interval) between the two sampling locations for each given metabolite at 2 h, 4 h and 6 h time points (SI Table 2).

## Supplementary information

Supplementary Information

## Data Availability

The data generated from the current study are available from the corresponding author on request.
