# Supplementary material for: Label-free time- and space-resolved exometabolite sampling of growing plant roots through nanoporous interfaces
Source: Sci Rep. 2019 Jul 16;9:10272. doi: 10.1038/s41598-019-46538-5 (PMC6635491; doi:10.1038/s41598-019-46538-5)
Supplement: Supplementary file 1 — Supplementary Information [file 41598_2019_46538_MOESM1_ESM.docx]

***Supporting Information:***

**Label-free time- and space-resolved exometabolite sampling of growing plant roots through nanoporous interfaces**

Damith E.W. Patabadige^1^, Larry J. Millet^1^, Jayde A. Aufrecht^1,3^, Peter G. Shankles^1,3^, Robert F. Standaert^1,4^ , Scott T. Retterer^1-3*^, Mitchel J. Doktycz^1-3*^

*Correspondence to [doktyczmj@ornl.gov](mailto:doktyczmj@ornl.gov) or [rettererst@ornl.gov](mailto:rettererst@ornl.gov)

^1^ Biosciences Division, Oak Ridge National Laboratory, PO Box 2008 MS 6445, Oak Ridge, TN 37831-6445

^2^ The Center for Nanophase Materials Sciences, Oak Ridge National Laboratory, PO Box 2008 MS 6445, Oak Ridge, TN 37831-6445

^3^ The Bredesen Center, University of Tennessee-Knoxville, Knoxville, TN

^4^ Shull Wollan Center, Oak Ridge National Laboratory, PO Box 2008 MS 6445, Oak Ridge, TN 37831-6445

***Device Assembly***

*
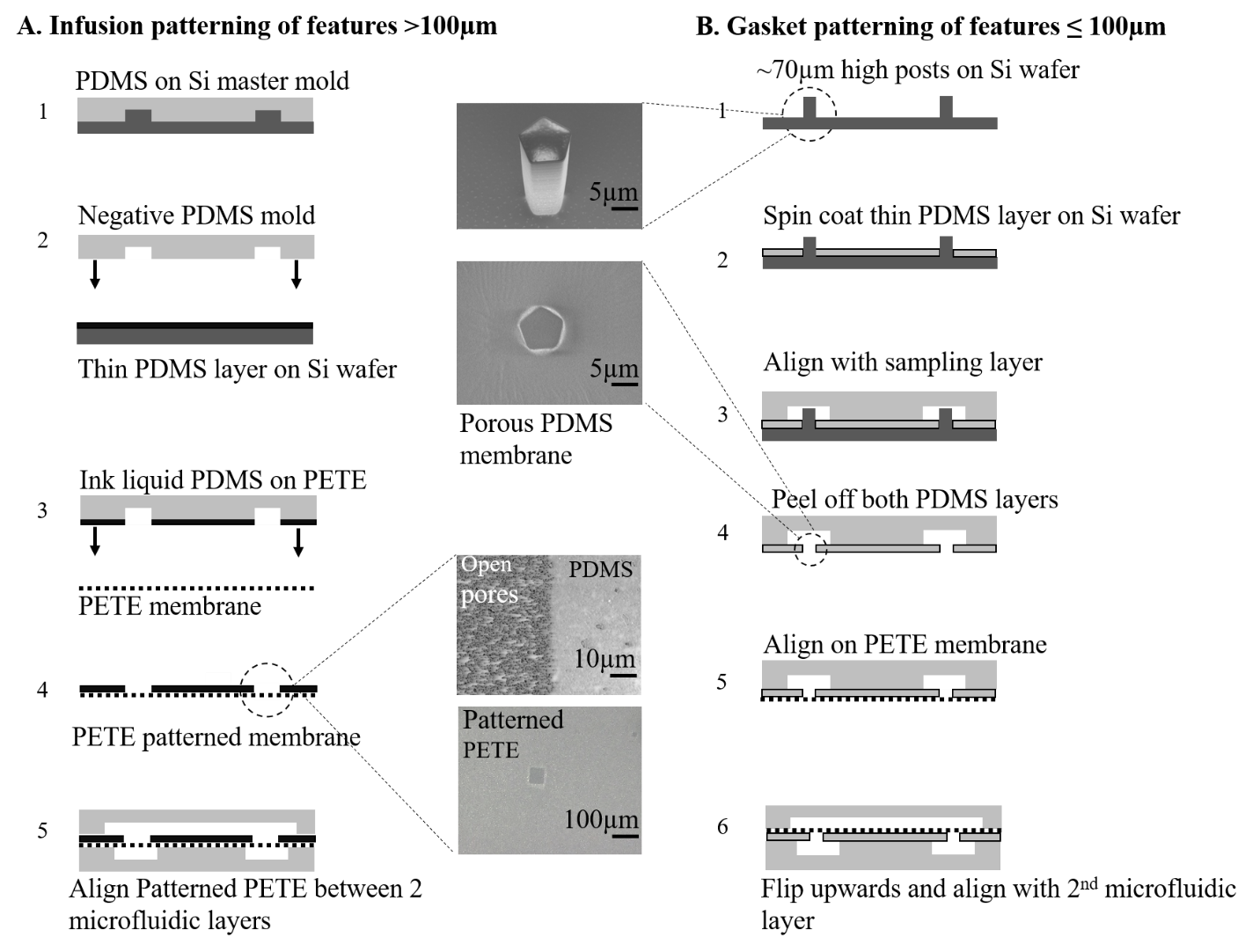
*

**SI Figure 1**: Schematic of the steps for integrating the PETE membrane between the PDMS-based sample microenvironment and metabolite sampling layers. **(A)** Process flow for creating pattern-infused nanoporous membranes within microfluidic systems. Open and closed regions of nanopores are indicated by areas without and with infused PDMS in the membrane, respectively. **(B)** Process flow for gasket patterning of the nanoporous PETE membrane. This approach provides for smaller sampling and dosing windows. Shown are ~70µm high pentagonal silicon posts (middle, top) on a wafer that is used to create a PDMS gasket.

*Bonding microfluidic layers and nanoporous interface*

The three-layer nano-enabled sampling platform was created using two microfluidic layers and a patterned nanoporous interface. First, the top and bottom microfluidic layers were plasma-treated to activate the PDMS surfaces for covalent PDMS-to-PDMS bonding. The bottom layer (metabolite collection fluidic layer) was carefully placed on the patterned membrane such that the sampling areas were aligned (using the inverted microscope) with the fluidics and each dosing area was individually addressable. Next, bonded layers were turned up-side down such that sampling areas were facing upwards. Then, the top-layer (sample culture fluidic layer) was aligned such that nanofluidic interface was sandwiched between two layers. Finally, the device was placed in 40 °C oven overnight to improve the bonding and sealing between PDMS layers and the patterned membrane.

The four-layer device with porous PDMS membrane was assembled similar to the previously described procedure except for the additional step of PDMS membrane attachment. PDMS membrane was first attached to the layer with metabolite collection channels. Then the un-pattern membrane was sandwiched between porous PDMS membrane and sample culture fluidic layer.

*Fluid pumping for sample collection*

Fluid-in


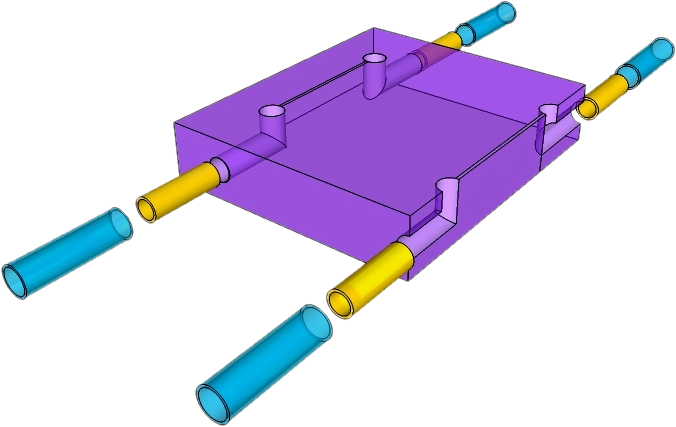


Fluid-out

Sampling channel

Bottom microfluidic layer

**SI Figure 2**: Schematic of sample collection and fluid pumping in sampling layer (bottom-layer). A hole was punched halfway through from the top (perpendicular to the channel), followed by punching 2^nd^ hole from the parallel direction to make right angle fluidic reservoir

**Identified metabolites of wheat root**

**Table S1**: Temporal variation of identified metabolites, their retention time and peak signatures

| Metabolite | Retention time (min)^a^ | Molecular- ion peak (m/z)^b^ | Extracted Ion Peak Area | | | | |
| --- | --- | --- | --- | --- | --- | --- | --- |
|  |  |  | After 2h | After 4h | After 7h | After 10h | After 16h |
| Palmitic acid | 12.35 | 313 | 0 | 0 | 0 | 0 | 227080 |
| Stearic acid | 13.25 | 117 | 0 | 0 | 0 | 0 | 183952 |
| Glycerol | 7.88 | 205 | 43672 | 36154 | 58049 | 67890 | 131731 |
| Phosphoric acid | 7.93 | 299 | 0 | 0 | 0 | 0 | 38976 |
| Alanine | 6.62 | 116 | 22908 | 26200 | 35660 | 27028 | 31792 |
| 5-Oxoproline | 9.59 | 156 | 9316 | 19068 | 35100 | 11756 | 20252 |
| Glycine | 8.17 | 174 | 8480 | 8132 | 14228 | 9408 | 13448 |
| Isoleucine | 8.06 | 158 | 3468 | 3396 | 6048 | 5160 | 3844 |
| Valine | 7.50 | 144 | 1724 | 2572 | 5688 | 1496 | 3100 |
| Sucrose | 15.09 | 361 | 0 | 8320 | 17748 | 632 | 2532 |
| Serine | 8.54 | 204 | 3256 | 4864 | 7976 | 3088 | 6896 |
| Tryptophan | 13.28 | 202 | 264 | 552 | 9176 | 1432 | 1936 |
| Glutamine | 10.82 | 156 | 0 | 288 | 368 | 0 | 1548 |
| Malic acid | 9.36 | 233 | 1068 | 784 | 22624 | 5680 | 1044 |
| Threonine | 8.72 | 218 | 548 | 1240 | 1480 | 568 | 620 |
| Proline | 8.09 | 142 | 1292 | 1512 | 2984 | 684 | 2128 |
| Thiourea | 9.29 | 171 | 88 | 81 | 373 | 654 | 414 |
| Phenylalanine | 10.25 | 218 | 0 | 320 | 8588 | 0 | 368 |
| Arginine | 11.30 | 157 | 244 | 0 | 300 | 0 | 0 |
| Cysteine* | 9.80 | 220 | 0 | 0 | 0 | 0 | 0 |
| Asparagine* | 10.46 | 116 | 0 | 0 | 0 | 0 | 0 |

^a^ Samples was analyzed by Agilent 5975 triple quadrupole GC-MS system with HP-5ms column

^b^ Analytes were derivatized with MSTFA and major peaks are associated with derivatized analytes, where, ‘major peak’ is the ‘molecular-ion peak’ of the derivaltized analyte and not necessarily the peak with highest intensity.

*These compounds were not present in the metabolite collection layer. However, they were detected in culture fluidic layer.

Total collection of identified metabolites recovered through the membrane (200 µm x 200 µm sampling area). Time=0 was set after plant grow 40 h in the Murashige-Skoog media (hydroponic plant nutrient solution). Temporal variation of the detected metabolites was monitored over the course of 16 h from the starting point. Concentration was measured as peak area produced by a given analyte.

**Direct Sampling of Metabolites (Control experiment)**

Sucrose

Valine

5-Oxoproline

Alanine

**SI** **Figure 3**: Concentration variation of selected metabolites over time for two distinct sample locations. Samples (20 µL each) were drawn using a pipette from the locations close to the root tip (location 1) and root base (location 2). Error bars represent standard deviation of three experiments. The Student t-test indicate that the metabolite concentrations at the two sampling locations are not significantly different at P<0.05 (95% confidence interval) for each given time point except 5-oxoproline at the 6h time point.

**Table S2***:*

1. Student t-test and statistical significance of each metabolite. A “-” indicates insufficient data for the statistical test.

| Analyte | Alanine | | |
| --- | --- | --- | --- |
| Time point (h) | 2 | 4 | 6 |
| t-value | 2.2272 | 1.8785 | 2.9736 |
| Critical value | 3.182 | 3.182 | 2.776 |
| Significantly different at p<0.05 | No | No | Yes |

| Analyte | 5-Oxoproline | | |
| --- | --- | --- | --- |
| Time point (h) | 2 | 4 | 6 |
| t-value | 4.8245 | 4.4513 | 7.6235 |
| Critical value | 3.182 | 3.182 | 2.776 |
| Significantly different at p<0.05 | Yes | Yes | Yes |

| Analyte | Isoleucine | | |
| --- | --- | --- | --- |
| Time point (h) | 2 | 4 | 6 |
| t-value | -0.5276 | -0.1088 | 0.5763 |
| Critical value | 3.182 | 3.182 | 2.776 |
| Significantly different at p<0.05 | No | No | No |

| Analyte | Sucrose | | |
| --- | --- | --- | --- |
| Time point (h) | 2 | 4 | 6 |
| t-value | - | - | 6.5904 |
| Critical value | - | - | 2.776 |
| Significantly different at p<0.05 | - | - | Yes |

1. Student t-test and statistical significance of each metabolite of control experiment

| Analyte | Alanine | | |
| --- | --- | --- | --- |
| Time point (h) | 2 | 4 | 6 |
| t-value | -0.2495 | 0.3781 | -0.1316 |
| Critical value | 2.776 | 2.776 | 2.776 |
| Significantly different at p<0.05 | No | No | No |

| Analyte | 5-Oxoproline | | |
| --- | --- | --- | --- |
| Time point (h) | 2 | 4 | 6 |
| t-value | 1.8966 | -0.6226 | -2.9609 |
| Critical value | 2.776 | 2.776 | 2.776 |
| Significantly different at p<0.05 | No | No | Yes |

| Analyte | Valine | | |
| --- | --- | --- | --- |
| Time point (h) | 2 | 4 | 6 |
| t-value | 0.1354 | -1.1448 | 0.1556 |
| Critical value | 2.776 | 2.776 | 2.776 |
| Significantly different at p<0.05 | No | No | No |

| Analyte | Sucrose | | |
| --- | --- | --- | --- |
| Time point (h) | 2 | 4 | 6 |
| t-value | - | 0.2262 | -0.1436 |
| Critical value | - | 2.776 | 2.776 |
| Significantly different at p<0.05 | No | No | No |

**Table S3***:* Diffused fractions % of selected metabolites through 50x50 µm sampling window at different concentrations after 8 h. A “-” indicates that the metabolite was not detected.

**Alanine**

| Sample culture fluidic layer (µM) | Metabolite collection fluidic layer (nM) | % C/C_0_ fraction diffused |
| --- | --- | --- |
| 2100.00 | 1472.00 | 0.17 |
| 250.00 | 140.40 | 0.14 |
| 50.00 | 28.43 | 0.14 |
| 20.00 | 13.66 | 0.17 |
| 5.00 | 2.98 | 0.15 |
| 1.00 | 1.64 | 0.16 |

**Valine**

| Sample culture fluidic layer (µM) | Metabolite collection fluidic layer (nM) | % C/C_0_ fraction diffused |
| --- | --- | --- |
| 1500.00 | 1337.28 | 0.22 |
| 160.00 | 131.63 | 0.20 |
| 40.00 | 31.32 | 0.19 |
| 15.00 | 12.08 | 0.20 |
| 3.50 | 2.28 | 0.16 |
| 1.00 | 1.58 | 0.15 |

**Isoleucine**

| Sample culture fluidic layer (µM) | Metabolite collection fluidic layer (nM) | % C/C_0_ fraction diffused |
| --- | --- | --- |
| 1600.00 | 997.22 | 0.15 |
| 150.00 | 88.48 | 0.15 |
| 30.00 | 16.57 | 0.14 |
| 15.00 | 9.27 | 0.15 |
| 10.00 | 4.80 | 0.12 |
| 1.60 | 1.53 | 0.10 |

**Phenylalanine**

| Sample culture fluidic layer (µM) | Metabolite collection fluidic layer (nM) | % C/C_0_ fraction diffused |
| --- | --- | --- |
| 1600.00 | 840.10 | 0.13 |
| 160.00 | 82.24 | 0.13 |
| 80.00 | 35.20 | 0.11 |
| 10.00 | 10.50 | 0.10 |
| 5.00 | - | - |
| 2.00 | - | - |

| Sample culture fluidic layer (µM) | Metabolite collection fluidic layer (nM) | % C/C_0_ fraction diffused |
| --- | --- | --- |
| 3200.00 | 1242.94 | 0.097 |
| 400.00 | 142.02 | 0.088 |
| 48.00 | 42.19 | 0.088 |
| 30.00 | - | - |
| 10.00 | - | - |
| 5.00 | - | - |

**Sucrose**

| Sample culture fluidic layer (µM) | Metabolite collection fluidic layer (nM) | % C/C_0_ fraction diffused |
| --- | --- | --- |
| 3200.00 | 1161.52 | 0.091 |
| 208.00 | 183.21 | 0.088 |
| 100.00 | - | - |
| 50.00 | - | - |
| 20.00 | - | - |
| 5.00 | - | - |

**Malic acid**
